# Social jetlag and metabolic control in non-communicable chronic diseases: a study addressing different obesity statuses

**DOI:** 10.1038/s41598-017-06723-w

**Published:** 2017-07-25

**Authors:** Maria Carliana Mota, Catarina Mendes Silva, Laura Cristina Tibiletti Balieiro, Walid Makin Fahmy, Cibele Aparecida Crispim

**Affiliations:** 1 Faculty of Medicine of the Federal University of Uberlândia, Minas Gerais, Brazil; 2 Department of Obstetrics, Hospital e Maternidade Municipal de Uberlândia, Uberlândia, Brazil

## Abstract

Social jetlag – a measure of disruption of the circadian system – has been linked to obesity, but its association with metabolic complications in non-communicable chronic diseases (NCCDs) is unknown in the literature. We examined the associations between social jetlag and obesity status and metabolic parameters among individuals with NCCDs. Patients (n = 792) with NCCDs (obesity, systemic arterial hypertension, type 2 diabetes mellitus or dyslipidaemia) attended clinics of the public health service of the city of Uberlândia, Minas Gerais State, Brazil. They were classified in three obesity statuses: non-obese: BMI < 30 kg/m^2^; metabolically healthy obese (MHO): BMI ≥ 30 kg/m^2^ and less than three high-risk biomarkers for metabolic syndrome; and metabolically unhealthy obese (MUO): BMI ≥ 30 kg/m^2^ and with high-risk values on three or more biomarkers for metabolic syndrome. After adjustments for confounding variables, social jetlag was positively associated with fasting glucose levels among all subjects (β = 0.08, p = 0.03) and MUO subjects (β = 0.32, p < 0.001). Patients with social jetlag (>1 h) presented a significant odds ratio (OR) of being overweight (OR = 2.0, confidence interval (CI) = 1.2–3.6, p = 0.006) and MUO (OR = 1.8, CI = 1.1–2.8, p = 0.01). These results suggest that social jetlag is associated with a higher risk of overweight and related metabolic complications in individuals with NCCDs.

## Introduction

The prevalence of obesity has increased rapidly over the past three decades, reaching epidemic levels worldwide^1, 2^. It has been well demonstrated that being overweight or obese are considered important risk factors for type 2 diabetes, systemic arterial hypertension, cardiovascular diseases, certain types of cancers and premature death^1, 3^. Interestingly, about one-third of all obese individuals seems to be more resistant to cardiovascular and metabolic consequences related to excess fat^4^. Despite increased adiposity, ‘metabolically healthy obese’ (MHO) subjects are characterized by a favourable metabolic profile: high levels of insulin sensitivity, a low prevalence of systemic arterial hypertension, favourable lipid and inflammation profiles^5^. In this context, a better understanding of the risk factors that can trigger or protect the comorbidities commonly associated with obesity could be important to avoid the poor prognosis of this disease^5–7^.

Although obesity has traditionally been thought to be caused by changes in diet and decreased levels of physical activity^8^, recent research has suggested that a number of alternative factors may be involved with the obesity genesis, such as circadian misalignment and sleep debt^9, 10^. In this sense, social jetlag – which describes the chronic jetlag-like phenomenon occasioned by work or study schedules and reflects a misalignment between an individual’s endogenous circadian clock and actual sleep times^11^ – seems to be associated with overweight^10^ and metabolic parameters^12^. In addition, previous studies have shown that social jetlag is associated with unhealthy behaviours: smoking, mental distress, alcohol use^11, 13^ and lower level of physical activity^14^.

To date, the associations between social jetlag and metabolic parameters have predominantly been studied in the general population and without a focus on individuals with non-communicable chronic diseases (NCCDs). In one of these studies, Wong *et al*.^12^, evaluated middle-aged adults (n = 490, 42.7 ± 7.4, 47% men) and identified positive associations between social jetlag and serum triglyceride levels, fasting insulin and insulin resistance in the Homeostatis Model Assessment (HOMA-IR). In addition, Parsons *et al*.^15^, demonstrated that social jetlag in young adults (n = 1037; 52% males, 38 years) was a risk factor for higher than recommended glycated haemoglobin (HbA1c) levels (>5.7%) and a higher risk of being metabolically unhealthy obese (MUO). In a few studies that have analysed patients with metabolic abnormalities, social jetlag has been significantly associated with HbA1c levels in type 1 diabetes individuals (n = 80; 46% female)^16^. However, no associations have been found between social jetlag and parameters related to glycaemic control in type 2 diabetics^17^ or pre-diabetic individuals^18^. Thus, this is a topic still little explored that demonstrates the need to carry out new studies.

Accordingly we hypothesized that social jetlag is negatively associated with excessive weight (overweight/obesity) and metabolic parameters among individuals with NCCDs and those with social jetlag are at increased risk of overweight or obese or MUO. The objective of this study was to analyse the associations between social jetlag and metabolic parameters among individuals with NCCDs, addressing different obese statuses.

## Results

### Participant characteristics

This study included 792 individuals presented in Table 1. Most participants were women (n = 581; 73%), married (n = 401; 51%) and had less than 12 years of schooling (n = 507; 64%). Forty-two per cent of the patients (n = 333) were day workers and 32% (n = 256) were retired. According to reported health behaviours, 37% (n = 294) engaged in physical activity and the median weekly time spent on physical activity by these subjects was 180 minutes [120–300 minutes].Twelve percent of individuals (n = 96) reported being smokers, and the median consumption of alcoholic beverages per week was 2.0 (0.75–6.0) among those who reported usually consuming some type of alcoholic beverage (28%, n = 220; median of servings *per* week = 2.0 [0.75–6.0]).

**Table 1:** Demographics, employment status, anthropometric, health behaviors, physical activity, sleep, social jetlag and metabolic variables according to obesity status (n = 792)

Variables	All (n = 792)	Non-obese (n = 369)	MHO (n = 252)	MUO (n = 171)	p*
Age (years)	55.9 ± 12.4	55.6 ± 12.3	55.4 ± 13.4	56.4 ± 13.6	0.17
Female (%)	581 (73.0)	269 (73.1)	189 (75.3)	122 (71.3)	0.65
Marital status – Married (%)	401 (51.0)	188 (51.0)	131 (52.0)	82 (48.5)	0.42
Family income – (U$ 553.0)	504 (63.0)	235 (63.7)	151 (60.0)	118 (69.4)	0.36
Education – <= 12 years	507 (64.0)	231 (62.6)	165 (65.4)	123 (72.0)	0.37
Employment status					
Day workers (%)	333 (42.0)	144 (38.7)	96 (40.0)	68 (39.6)	0.88
Retired (%)	256 (32.0)	132 (36.3)	91 (33.6)	57 (33.5)	
Hours per week	40.8 ± 8.9	41.0 ± 6.7	39.4 ± 7.9	42.4 ± 7.1	0.12
Ex-night worker	37 (5.0)	22 (2.8)	7 (4.7)	8 (6.0)	0.17
Health behaviours					
Smoking status – Yes (%)	96 (12.0)	55 (15.0)	27 (10.7)	14 (8.1)	0.05
Alcohol intake – Yes (%)	220 (28.0)	103 (28.0)	75 (29.7)	41 (24.0)	0.26
Alcohol – Servings/week	2.0 [0.75–6.0]	2.0 [0.5–7.0]	2.0 [1.0–5.2]	2.3 [0.5–5.0]	0.86
Physical activity (PA) – Yes (%)	294 (37.0)	134 (36.4)	93 (37.0)	67 (39.1)	0.82
Minutes of PA/week^**Χ**^	180 [120–300]	190 [120–300]	170 [120–300]	180 [120–290]	0.31
Chronic diseases					
Type 2 diabetes mellitus (TD2) (%)	257 (33.0)	111 (30.0)	57 (22.6)	89 (52.3)	**<0**.**001**
Arterial hypertension (SAH) (%)	526 (66.4)	223 (63.0)	174 (69.0)	129 (75.4)	**0**.**02**
Dyslipidemia (%)^⚲^	324 (41.0)	148 (40.0)	89 (35.4)	87 (50.8)	**<0**.**006**
Time of diagnosis of TD2^λ^	5.0 [3.0–10.0]	6.0 [3.0–10.0]	9.5 [4.0–16.0]	6.0 [3.0–10.0]	0.63
Time of diagnosis of SAH^λ^	10 [5.0–15.0]	10.0 [5.0–15.0]	8.0 [2.0–15.0]	10.0 [5.0–18.0]	0.15
Time of diagnosis of dyslipidaemia^λ^	5.0 [2.0–10.0]	4.0 [2.0–8.0]	4.0 [2.0–10.0]	5.0 [3.0–10.0]	0.13
Anthropometric					
BMI (kg/m^2^)	30.0 ± 6.3	25.1 ± 0.2^a^	33.4 ± 0.2^b^	33.6 ± 0.3^b^	**<0**.**001**
Abdominal obesity (%)^Ω^	564 (71.0)	173 (47.0)	224 (89.0)	167 (98.0)	**<0**.**001**
High neck circumference (%)^Φ^	518 (65.0)	155 (42.2)	209 (83.2)	154 (90.0)	**<0**.**001**
Food intake					
Kcal (kcal/day)	1467.5 [1212.3–1796.8]	1439.5 [1199.5–1764.8]	1496.4 [1221.5–1857.3]	1511.1[1254.0–1860.0]	0.34
Carbohydrate (g/day)	174.1 [137.0–215.1]	170.0 [136.1–213.6]	181.2 [136.3–226.5]	174.1 [137.0–215.1]	0.21
Protein intake (g/day)	73.9 [55.7–98.8]	73.1 [56.4–94.6]	77.0 [55.0–104.2]	75.4 [56.3–102.7]	0.58
Fat intake (g/day)	51.0 [35.2–69.0]	51.0 [34.5–67.3]	52.4 [36.4–73.2]	51.0 [35.2–70.0]	0.74
Kcal after 9 p.m. (kcal/day)	0 [0–165.4]	0 [0–204.8]	0 [0–138]	0 [0–130.0]	0.70
Circadian					
Chronotype – MSFsc (h)^Ψ^	02:54 [02:06–03:48]	02:48 [02:00–02:42]	02:59 [2:07–03:49]	02:55 [02:12–03:54]	0.72
Morning (%)	633 (79.0)	296 (80.2)	197 (78.1)	139 (81.2)	0.83
Intermediate (%)	77 (9.7)	33 (9.0)	29 (11.5)	15 (8.8)	
Late (%)	83 (10.4)	40 (10.8)	26 (10.3)	17 (10.0)	
Bedtime weekday (h)^δ^	22:20 [21:20–23:20]	22:20 [21:49–23:00]	22:22 [21:20–23:30]	22:24 [21:13–23:25]	0.93
Bedtime weekend (h)^δ^	22:50 [21:50–24:00]	22:48 [21:37–23:00]	22:55 [21:52–23:55]	22:49 [21:50–23:51]	0.35
Waketime weekday (h)^δ^	06:00 [05:30–07:00]	06:00 [05:30–07:00]	06:22 [06:00–07:00]	06:00 [05:30–07:30]	0.43
Waketime weekend (h ^δ^	07:00 [06:00–08:30]	07:00 [06:00–08:30]	07:00 [06:00–08:30]	07:00 [06:00–08:00]	0.48
Sleep duration weekday (h)	07:30 [06:30–09:00]	07:30 [07:00–09:00]	07:30 [06:30–09:00]	07:00 [06:30–09:00]	0.32
Sleep duration weekend (h)	08:00 [07:00–09:00]	08:00 [07:00–09:00]	08:00 [07:00–09:00]	08:00 [07:30–09:00]	0.36
Mean sleeping duration (h)	07:29 ± 01:50	07:36 ± 01:47	07:25 ± 02:05	07:23 + 01:50	0.65
Social jetlag (h)	00:30 [00:00–01:00]	00:30 [00:00–01:00]	0:30 [0:00–01:00]	0:25 [0:00–01:15]	0.97
Social jetlag <= 1 h (%)	524 (66.0)	279 (75.6)	193 (76.6)	126 (73.6)	0.42
Social jetlag >1 h and <2 h (%)	117 (14.0)	48 (13.0)	39 (15.4)	30 (17.4)	
Social jetlag > = 2 h (%)	77 (10.0)	42 (11.3)	20 (8.0)	15 (9.0)	

*One-way ANOVA and Tukey post hoc analyses were performed for normally distributed variables. When the variables were not normally distributed, Kruskal–Wallis tests were used. Variables with significant values in the Kruskal–Wallis test were tested by Dunn’s test with a correction of alpha via Bonferroni’s method. Values are presented as mean and SD for normally distributed data or as median (interquartile range) for non-normally distributed data. ^Ψ^Chronotype (MSF) was derived from time of mid-sleep on free days (weekend), with further correction for calculated sleep debt – the difference between average sleep duration at weekends and on weekdays. ^δ^Time is presented in 24-h clock time. ^⚲^Diagnosis of hypercholesterolaemia, hypertriglyceridaemia or reduced HDL-C. ^Ω^Waist circumference ≥ 102 cm for men and ≥ 88 cm for women were considered abdominal obesity. ^Φ^Neck circumference ≥ 39 cm for men and ≥ 35 cm for women were considered high. ^λ^Only those reported to have type 2 diabetes mellitus, systemic arterial hypertension or dyslipidaemia were included. ^Χ^Only those reported to perform physical activities. BMI: Body mass index. MHO: Metabolically healthy obese. MUO: Metabolically unhealthy obese.

Prevalence of type 2 diabetes (p < 0.001), systemic arterial hypertension (p = 0.02) and dyslipidaemia (p < 0.006) was higher among the MUO (Table 1). As expected, body mass index averages (p < 0.001) and prevalence of abdominal obesity (p < 0.001) and high neck circumference (p < 0.001) was also higher among the MUO and MHO groups when compared with the non-obese group. Regarding the circadian data, a total of 24% (n = 192) had a social jetlag degree higher than 1 h, and 10% (n = 77) greater or equal to 2 h (Table 1). No significant difference was found when comparing the circadian variables between the different obesity statuses.

A total of 46.5% (n = 369) were classified as non-obese, 32% (n = 252) were MHO and 21.5% (n = 171) were MUO. No significant difference was found between the socio-demographic variables and those related to health behaviours when comparing the different obesity statuses (Table 1).

### Associations between social jetlag and anthropometric, metabolic parameters and blood pressure

Multiple linear regression analysis associating social jetlag and anthropometric variables, metabolic parameters and blood pressure are shown in Table 2. After adjustments for possible confounding variables, social jetlag was positively associated with fasting glucose levels among all subjects (β = 0.08, p = 0.03, r^2^ adjusted = 0.11) and also among MUO subjects (β = 0.32, p < 0.001, r^2^ adjusted = 0.13). After adjustments for confounders, social jetlag was associated with total cholesterol (β = 0.19, p = 0.04, r^2^ adjusted = 0.01) and triglycerides levels (β = 0.33, p = 0.001, r^2^ adjusted = 0.23) among MUO subjects.

**Table 2:** Associations between social jetlag and anthropometric, metab olic parameters and blood pressure according to obesity status (n = 792)

	All (n = 792)		Non-obese (n = 369)		MHO (n = 252)		MUO (n = 171)	
	β	p	β	p	β	p	β	p
BMI (Kg/m^2^)	0.03	0.38	0.02	0.65	−0.06	0.38	−0.03	0.53
Waist circumference, cm	0.01	0.84	0.02	0.69	0.01	0.98	0.08	0.21
Neck circumference, cm	−0.01	0.88	−0.01	0.76	−0.03	0.68	0.03	0.62
Weight gain, kg	−0.04	0.37	0.04	0.76	−0.08	0.44	−0.01	0.90
Fasting glucose, mg/dL	0.08	**0**.**03** ^**ΦΧ**^	0.08	0.14	−0.05	0.63	0.32-	**<0**.**001** ^**ΦΩ**^
Glycated haemoglobin, %	0.01	0.82	−0.02	0.68	0.10	0.53	0.01	0.82
Total cholesterol, mg/dL	0.02	0.59	0.02	0.72	0.01	0.89	0.19	**0**.**04** ^**δψ**^
HDL-c, mg/dL	0.05	0.15	0.09	0.12	0.05	0.60	0.13	0.16
LDL-c, mg/dL	−0.01	0.80	−0.04	0.48	0.01	0.87	0.08	0.39
Triglycerides, mg/dL	0.04	0.26	0.07	0.27	−0.08	0.30	0.33	**0**.**001**
Systolic BP, mm Hg	0.01	0.65	−0.04	0.49	0.13	0.23	0.03	0.72
Diastolic BP, mm Hg	0.01	0.91	0.01	0.81	−0.08	0.44	0.07	0.42

^Φ^Multivariate linear regressions analysis adjusted for age, sex, family income, time of diagnosis of type 2 diabetes mellitus, insulin use, minutes of physical activity per week, mean of sleep time. ^δ^Multivariate linear regressions analysis adjusted for age, sex, family income, time of diagnosis of type 2 diabetes mellitus, time of diagnosis of dyslipidaemia, insulin use, use of antidepressants and mean of sleep time.  Multivariate linear regressions analysis adjusted for age, sex, family income, employment status, time of diagnosis of dyslipidaemia and use of sleeping pills. ^Χ^r^2^ adjusted = 0.11; ^Ω^r^2^ adjusted = 0.13; ^ψ^r^2^ adjusted = 0.01;  r^2^adjusted = 0.23. MHO: Metabolically healthy obese. MUO: Metabolically unhealthy obese.

The results of logistic regression showed that the crude and the adjusted model indicated a higher risk of being overweight (BMI ≥ 25 kg/m^2^) for individuals that presented social jetlag (>1 h) (odds ratio [OR] = 2.0, confidence intervals [CI] = 1.2–3.6, p = 0.006] in comparison with those without social jetlag (≤1 h) (Table 3). The adjusted model also showed a risk of being an MUO (OR = 1.8, CI = 1.1–2.8, p = 0.01) for individuals that presented social jetlag (>1 h) in comparison with those without social jetlag (≤1 h).

**Table 3:** Odds ratio (OR) for comparison of effects of social jetlag (>1 h) versus no social jetlag (≤1 h) on overweight, obese or metabolically unhealthy obesity (n = 792)

	Overweight^⚲^		Obese^Χ^		MUO^δ^	
	OR (95% CI)	p	OR (95% CI)	p	OR (95% CI)	p
Crude	1.8 (1.2–2.7)	**0**.**005**	1.0 (0.8–1.5)	0.60	1.2 (0.8–1.7)	0.30
Adjusted model	2.0 (1.2–3.6)^ψ^	**0**.**006**	1.1 (0.7–1.6)	0.53	1.8 (1.1–2.8)^Φ^	**0**.**01**

^ψ^Multivariate logistic regressions analysis adjusted model for age, sex, employment status, insulin use, use of sleeping pills, use of antidepressants and time of diagnosis of type 2 diabetes mellitus, systemic arterial hypertension or dyslipidaemia. ^Φ^Multivariate logistic regressions analysis adjusted model for age, sex, insulin use, use of sleeping pills, minutes of physical activity per week, and time of diagnosis of type 2 diabetes mellitus, systemic arterial hypertension or dyslipidaemia. ^⚲^Overweight (BMI ≥ 25 kg/m^2^). ^Χ^Obese (BMI ≥ 30 kg/m^2^). ^δ^MUO (BMI ≥ 30 kg/m^2^ and high-risk values on three or more biomarkers for metabolic syndrome). CI: confidence intervals. MUO: Metabolically unhealthy obese. OR: odds ratio.

## Discussion

This study evaluated the relationship between social jetlag, obesity, metabolic parameters and blood pressure among individuals with NCCDs. A higher odds of being overweight and an MUO was found in individuals with social jetlag, even after adjustment for factors that may also influence the development of excess weight and its metabolic complications. We also found that social jetlag was positively associated with fasting glucose when all participants were analysed, regardless of the obesity status, as well as when we analysed only MUO. Furthermore, we found that social jetlag was associated with total cholesterol and triglyceride levels in MUO individuals. These data confirm our initial hypothesis – that circadian desynchronization measured by social jetlag is associated with excessive weight and metabolic parameters among individuals with NCCDs. To the best of our knowledge, this is the first study showing that social jetlag is associated with anthropometric and metabolic risk factors in individuals with chronic diseases.

Regarding glycaemic control in individuals with metabolic abnormalities, Larcher *et al*.^16^, found in patients with type 1 diabetes a positive association between HbA1c and social jetlag (β = 0.012, p < 0.001); HbA1c levels were lower in patients with social jetlag below (≤49 min) versus above (>49 min) the median (7.7% and 8.7%, respectively; p = 0.011). However, in two studies also examining the associations between social jetlag and glycaemic control markers, conducted in pre-diabetic subjects^18^ and type 2 diabetes patients^17^, no significant associations were found between circadian misalignment and metabolic control. Two other studies involving the general population^12, 15^ revealed that social jetlag is related to a worse metabolic pattern, such as higher fasting plasma insulin, insulin resistance and higher LDL-c and triglyceride levels (p* < *0.05)^12, 15^. In this study, we included individuals with previous diagnoses of at least one NCCD (obesity, systemic arterial hypertension, type 2 diabetes mellitus and/or dyslipidaemia) and we found a positive association between social jetlag and fasting glucose among all and MUO subjects, as well as total cholesterol and triglyceride levels among MUO individuals (Table 2). This may have occurred because several physiological processes – such as glucose metabolism, core body temperature and blood pressure –can contribute to the risk of cardiovascular disease when they disrupt the intrinsic circadian rhythm^19^.

The circadian clock is a cell-autonomous molecular mechanism that is organized in a hierarchical structure on the organismal level^19^. Synchronization of the internal time with the external time is necessary for the maintenance of the synchronization of the body with the external time demands^19, 20^. Circadian clocks are synchronized (entrained) by environmental signals (*zeitgebers*), predominantly by sunlight^20^. In fact, social jetlag can lead to a delay in bedtime and this increases night-time light exposure, and this can lead to metabolic damage, such as reduced pancreatic β-cell compensation or reduced insulin sensitivity^21, 22^. The findings of the present study confirm that social jetlag is associated with metabolic problems related to obesity in patients with NCCDs. In view of the poor prognoses of these diseases, additional studies should confirm whether social jetlag control could prevent metabolic complications in these patients.

In our study, social jetlag was not related to HbA1c levels (Table 2), as also found in other studies^17, 18^. According to Anothaisintawee *et al*.^18^, a low prevalence of social jetlag, as found in this study (n = 164; 25%), may influence the association of this parameter with conditions of circadian misalignment. In fact, associations between HBA1c levels and social jetlag have been found in studies with a higher prevalence of social jetlag: 82% (n = 548/667)^15^ and 49% (n = 39/80)^16^. These factors show the need for further studies with different populations and over different periods investigating all of these parameters.

Although the great majority of obese individuals are predisposed to developing the comorbidities usually related to overweight, about 40% of obese subjects do not develop them^5^. The results found in the present study allow us to suppose that social jetlag may play a part in whether an obese person develops a comorbidity or not. This could explain why, interestingly, no associations were found between social jetlag and metabolic parameters in the non-obese and MHO groups (Table 2). Possibly the circadian misalignment of MUO individuals could elevated the sympathetic activation of the hypothalamo–pituitary–adrenal (HPA) axis, increasing levels of catecholamines and cortisol^23–25^. These hormonal changes could promote the development of an impaired glucose tolerance, insulin resistance, pancreatic β-cell dysfunction^23^ and atherogenic lipid profile^26, 27^. The findings of the present study show that social jetlag may actually be associated with metabolic problems related to obesity in patients with NCCDs. Given the poor prognosis of these diseases, additional studies should confirm whether social jetlag control could in some way prevent metabolic complications in these patients.

Another important finding of our study was the determination of the increased risk of being overweight and MUO among those with social jetlag (Table 3). An epidemiological study performed by Roenneberg *et al*.^10^, also showed that social jetlag was positively associated with weight increase in the overweight group (OR = 3.3 [95% CI: 2.5–4.3]). Parsons *et al*.^15^ also identified that individuals with higher social jetlag levels had an increased risk of being in the metabolically MUO group (OR = 1.8 [95% CI: 1.1–2.8, p = 0.01]). Because the circadian system organizes whole energy homeostasis, including food intake and caloric expenditure^28^, the disruption of the circadian clocks can lead to inadequate weight gain. Rutters *et al*.^24^ suggest that an activation of the HPA axis resulting from social jetlag may predispose to visceral obesity and other chronic diseases. Factors related to food intake – such as changes in meal distribution across the day^29^ and/or the type of food consumed^30^ – and physical activity pattern^14, 24^ could also explain weight change due to circadian disturbances^25^.

In this study, 46% (369/792) of the evaluated group were not obese and presented some chronic disease (type 2 diabetes mellitus: 30%; systemic arterial hypertension: 63.0%; dyslipidemia 40.0%). In this group (non-obese with NCCDs), no associations were found between metabolic parameters or blood pressure and social jetlag (Table 2), suggesting that obesity – especially among MUO individuals – can mediate the relationship between circadian misalignment and metabolic lack of control. The occurrence of NCCDs in Brazil has increased significantly in recent decades^31^, including in non-obese individuals. A study conducted by Cercato *et al*.^32^, found that although cardiovascular risk increased along with BMI, individuals with adequate weight (BMI = 18.5–24.9 kg/m^2^) or overweight (BMI = 25.0–29.9 kg/m^2^) had a prevalence of 19% and 25% of systemic arterial hypertension respectively, 4.5% and 11% of type 2 diabetes mellitus respectively and 54% and 59% of hypercholesterolemia respectively. These results indicate that non-obese individuals are not exempt from having NCCDs.

Previous studies have also found no associations between social jetlag and systemic blood pressure values^12, 24^, as we found in this study (Table 2). We also found no association between social jetlag and pressure levels in the groups evaluated. These results can be explained by the periodic monitoring of blood pressure and the use of hypotensive drugs, as occurred with the population in this study. These factors may favour the control of blood pressure and maintain blood pressure levels considered normal in individuals^33–35^. Nevertheless, there is some evidence that circadian misalignment may lead to an increase in blood pressure, for example a 3% increase in mean arterial pressure during short-term circadian misalignment (p = 0.001) found in a study under laboratory-controlled conditions^36^. Future studies with different population profiles and protocols are required to investigate whether circadian misalignment leads to increased blood pressure in individuals diagnosed with systemic arterial hypertension.

This study has some limitations. The cross-sectional design precludes causal inferences and warrants future prospective studies to extend the present findings. We also emphasize as a limitation the use of questionnaires which, although validated in other studies, are subjective and dependent on the memory and motivation of the participants. In particular, replacing the questionnaires with objective alternatives to obtain the sleep patterns could provide a more reliable basis for evaluating the degree of social jetlag. Also, although the average sleep time was considered as an adjustment factor for the analyses performed, we did not consider the sleep patterns of the nights immediately prior to the collection of blood; the generalization of data is also limited because of the relatively small number of volunteers and the inclusion of users of the Brazilian public health service, comprising approximately 70% of the population. Furthermore, some evidence suggests that fat distribution (more specifically visceral adipose and liver tissue mass) and inflammation play a role in the presence of metabolic and clinical conditions for metabolic syndrome in obese individuals^37–39^. These factors could not be evaluated in the present study. New studies that include these variables may lead to a better understanding of the influence of circadian misalignment on controlling metabolic parameters in individuals of different obese status.

In conclusion, our findings suggest that social jetlag negatively influences glycaemic and lipid control in patients with NCCDs, predisposing to a higher risk of obesity-related complications. Furthermore, social jetlag (>1 h) is associated with higher odds of being MUO, confirming that circadian misalignment may favour improper weight gain and metabolic syndrome. These findings highlight the importance of maintaining regular sleeping and waking times, balancing the biological and social demands and preferences of individuals who already have some type of metabolic dysfunction – as did the population of this study. This could avoid more severe metabolic complications in these individuals. Longitudinal studies should be performed to determine the real influence of social jetlag on the genesis of obesity and its complications.

## Materials and Methods

### Participants and ethics

The study was cross-sectional with volunteers who were attending the outpatient clinics of the public health service in the city of Uberlândia, Minas Gerais State, Brazil. Assessments were conducted from September 2015 to July 2016. The public health service offers outpatient health care to patients with chronic diseases, with systematic and periodic monitoring of metabolic parameters and also the provision of medicines to control these diseases. To be eligible to participate in the study, individuals had to have confirmed a pre-diagnosis of at least one of these chronic diseases: obesity, systemic arterial hypertension, type 2 diabetes mellitus or dyslipidaemia (hypercholesterolaemia, hypertriglyceridaemia or reduced HDL-C). Thus, the patient could be included regardless of the presence of overweight or obesity if they had one or more NCCDs used as criteria for inclusion in this study.

Individuals were excluded from the study if they: had time of diagnosis of chronic disease of less than one year (n = 2); were younger than 20 years old or over 80 (n = 7); were pregnant (n = 1); were shift workers (n = 2); had diseases or complications such as renal failure (n = 1), angina pectoris (n = 3), heart disease (8) and a history of heart attack (n = 7). During the course of the study eight patients refused to participate.

All methods were carried out in accordance with relevant guidelines and regulations. This study was approved by the Ethics Committee of the Federal University of Uberlândia (protocol n. 005464/2015). All volunteers signed a written informed consent form to participate in the study.

### Socio-demographic and health behaviours

The volunteers answered a questionnaire that assessed demographic aspects such as age, sex, years of education, marital status, family income and employment status. Participants were also asked about health behaviours related to physical activity, alcohol intake, smoking and the use of medicines.

### Metabolic parameters and blood pressure

Clinical and biochemical information was collected from the medical records of the volunteer, which included: systolic and diastolic blood pressure levels; lipid profile (low-density lipoprotein (LDL-c), high-density lipoprotein [HDL-c] and triglycerides); and glucose profile (fasting glucose and glycated haemoglobin). The most recent data taken up to six months prior to the study were collected and considered for the study. The collection, processing and analysis of blood samples for the determination of biochemical markers was performed in a single laboratory agreed to by the public health service and that follows widely established and consolidated standards. All volunteers were instructed to follow a period of fasting for 12 hours and at the time of collection, they were asked if this time was properly respected. Blood samples were collected on weekdays. The blood pressure measurement was performed by a doctor or nurse, also following all the protocols widely established to determine blood pressure levels^40^.

### Anthropometric variables

Weight was measured with a set of scales, to an accuracy of 0.1 kg (Welmy®). Height was measured with a stadiometer fixed to the wall, with an accuracy of 0.1 cm (Welmy®). Body mass index (BMI, kg/m²) was calculated^41^. The waist circumference (WC) was measured in agreement with the standard proposed by WHO^41^, as the minimum girth between the iliac crest and lower costal margin for the normal and overweight BMI and for obese individuals it was measured at umbilical level^42^. WC values ≥ 102 cm for men or ≥ 88 cm for women were considered elevated^41^. The neck circumference was measured by placing the tape around the neck, horizontally (Plano Frankfurt), with the individual standing erect. A neck circumference ≥39 cm for men and ≥35 cm for women were considered high^43^.

### Obesity status

The metabolic phenotype in MUO subjects is not homogeneous due to the use of discrepant definitions^5–7, 44, 45^ but in general uses parameters and criteria for the diagnosis of the metabolic syndrome. In this study we used the parameters and cut-off points proposed by Alberti el al^46^. Obesity status has been classified in three levels: non-obese: BMI < 30 kg/m^2^; MHO: BMI ≥30 kg/m^2^ and less than three high-risk biomarkers for metabolic syndrome; and MUO: BMI ≥ 30 kg/m^2^ and high-risk values on three or more biomarkers for metabolic syndrome. The biomarkers used were: 1) high waist circumference (≥88 cm for women, ≥102 cm for men); 2) high blood pressure (≥130/85 mm Hg); 3) low HDL-c (<50 mg/dL for women, <40 mg/dL for men); 4) high fasting glucose (≥100 mg/dL); and 5) high triglyceride levels (≥150 mg/dL)^46^.

### Sleep pattern, chronotype and social jetlag

Participants were asked to report their usual bedtimes and waking times on weekdays and weekends. Participants were asked: ‘What time do you usually go to sleep on weekdays?’; ‘How long (how many minutes as an average) do you stay awake in bed before you fall asleep (after lights off) on weekdays?’; ‘What time did you usually wake up on weekdays?’; ‘What time do you usually go to sleep at weekends?’; ‘How long (how many minutes as an,average) do you stay awake in bed before you fall asleep (after lights off) at weekends?’; ‘What time do you usually wake up at weekends?’ The bedtime on weekdays and at weekends was obtained whereas the time taken to fall asleep. These evaluations were performed by a team trained and experienced in sleep studies.

Sleep duration was computed using the weighted average of self-reported sleep duration, which considers both weekdays and weekends, using the formula: [(Reported current weekday sleep duration × 5) + (Reported current weekend sleep duration × 2)]/7^17^.

Chronotype was derived from the time of mid-sleep time on free days at the weekend (MSF), with a further correction for calculated sleep debt (MSFsc) - calculated as the difference between average sleep duration at the weekends and the average sleep in the week^47^. The chronotype was classified in: early types: MSFsc ≤ 3:59; intermediate types: MSFsc > 4:00 and < 4:59; and late types: MSFsc ≥ 5:00^10^. Social jetlag was calculated based on the absolute difference between mid-sleep time at weekends and on weekdays^11^.

### Food intake

Dietary intake was assessed by a single 24-hour food recall (24 h-FR) and an analysis of the energy intake and nutrient intake was performed using the Virtual Nutri Plus software®. The volunteers were instructed to provide as much detail as possible on the food and fluids consumed the previous day of interview, including brand names and recipes for home-cooked foods. Portion sizes were estimated using common household measurements such as cups, glasses, bowls, teaspoons, and tablespoons in addition to individual food items/units. The volunteers discussed their reported food intake with a qualified nutritionist, and the information was amended to include additional explanations and details, thus improving the accuracy of the information obtained. In this study the total intake of energy, carbohydrates and calorie intake after 9 p.m. were used as adjustment variables for the analyses performed.

### Statistical analysis

Initially, normality of the data was tested using the Kolmogorov–Smirnov test. The values are presented as the means and standard deviation, or as median [interquartile range] for non-normally distributed data. To characterize participants according to obesity status, one-way ANOVA and Tukey post hoc analyses were performed for normally distributed variables. When the variables were not normally distributed, Kruskal–Wallis tests were used. Variables with significant values in the Kruskal–Wallis test were tested by Dunn’s test with a correction of alpha via Bonferroni’s method.

To determine whether social jetlag was associated with anthropometric and metabolic parameters and blood pressure, linear regression was performed while controlling for confounding factors (age, sex, family income, employment status, time of diagnosis of the disease, mean sleep time, minutes of physical activity per week, use of insulin, antidepressants and/or sleeping pills). Variables that correlated with anthropometric or metabolic parameters or blood pressure in the Pearson or Spearman’s correlation (r > 0.20) were subjected to stepwise multivariate linear regression. To remove the influence of multicollinearity from the multiple regression model, tolerance and variance-inflation factors (VIFs) were determined and variables with a tolerance < 0.1 or VIF > 10.0 were removed from the model. Univariate and multivariate logistic regression were used to predict the risk of being overweight (BMI ≥ 25 kg/m²), obese (BMI ≥ 30 kg/m²) or MUO among those who had social jetlag (>1 h). Results were expressed as the odds with 95% CI. Variables with p-values < 0.20 entered in multiple regression models 1 and 2. In model 1, all variables were analysed together. In Model 2, a backward stepwise procedure was conducted. All statistical analyses were performed with the SPSS version 20.0 (SPSS Inc., Chicago, IL) and p < 0.05 was considered to be statistically significant.
